# MultiDomainBenchmark: a multi-domain query and subject database suite

**DOI:** 10.1186/s12859-019-2660-5

**Published:** 2019-02-14

**Authors:** Hyrum D. Carroll, John L. Spouge, Mileidy Gonzalez

**Affiliations:** 1 TSYS School of Computer Science, Columbus State University, 4225 University Avenue, Columbus, 31907 GA USA; 2 National Center for Biotechnology Information, Bethesda, National Institutes of Health, 8600 Rockville Pike, Bethesda, 20894 MD USA

**Keywords:** Multi-domain, Benchmark, Query and subject

## Abstract

**Background:**

Genetic sequence database retrieval benchmarks play an essential role in evaluating the performance of sequence searching tools. To date, all phylogenetically diverse benchmarks known to the authors include only query sequences with single protein domains. Domains are the primary building blocks of protein structure and function. Independently, each domain can fulfill a single function, but most proteins (>80% in Metazoa) exist as multi-domain proteins. Multiple domain units combine in various arrangements or architectures to create different functions and are often under evolutionary pressures to yield new ones. Thus, it is crucial to create gold standards reflecting the multi-domain complexity of real proteins to more accurately evaluate sequence searching tools.

**Description:**

This work introduces MultiDomainBenchmark (MDB), a database suite of 412 curated multi-domain queries and 227,512 target sequences, representing at least 5108 species and 1123 phylogenetically divergent protein families, their relevancy annotation, and domain location. Here, we use the benchmark to evaluate the performance of two commonly used sequence searching tools, BLAST/PSI-BLAST and HMMER. Additionally, we introduce a novel classification technique for multi-domain proteins to evaluate how well an algorithm recovers a domain architecture.

**Conclusion:**

MDB is publicly available at http://csc.columbusstate.edu/carroll/MDB/.

**Electronic supplementary material:**

The online version of this article (10.1186/s12859-019-2660-5) contains supplementary material, which is available to authorized users.

## Background

Genetic sequence database searching is a foundational tool in bioinformatics commonly used to make new discoveries, guide annotation, and direct downstream analysis, among many other tasks. Therefore, the performance of database searching tools is crucial to high quality results in many biomedical applications. Benchmarking such tools provides a systematic comparison to aid developers and researchers to understand the strengths of each tool. Here, we introduce the first phylogenetically diverse benchmark of multi-domain protein sequences.

Decades ago, the first benchmarks for genetic sequence database retrieval were comprised of single domain sequences. With less supporting evidence then we now enjoy, benchmark designers used just single domain sequences to provide a robust standard and to simplify homology evaluation. Databases such as Pfam [1], SCOPe [2], and others have been used by developers and researchers as benchmarks for over two decades [3]. Pfam is a large, partially curated database of protein families relying on hidden Markov models to guide homology designations. Many projects have leveraged the quality and breadth of Pfam, including RefProtDom [4]. RefProtDom applied several quality filters to Pfam entries, namely: long domain length, broad taxonomic diversity, and the availability of a structure. Although RefProtDom incorporates multiple domains in the target sequences, all its queries have a single domain. The SCOPe team has explicitly produced a subset of data known as the ASTRAL compendium [5]. For many years, developers and researchers have benchmarked sequence searching tools using ASTRAL [6–11]. Like SCOPe, ASTRAL is limited to high quality, but easily crystallizable and well-characterized proteins in PDB [12]. However, both SCOPe and ASTRAL restrict their homology annotations to single domain relationships to keep relationships simple and well-defined.

Other databases have also been used for benchmarking sequence searching tools. The OMA (“Orthologous MAtrix”) database [13] provides millions of orthologous pairs for over 2000 genomes. Terrapon et al. used OMA to determine homology between two sequences based on whether each contained at least one domain instance that is part of an orthologous pair [14]. While OMA naturally supports annotations on multiple domains and provides millions of orthologous pairs, it does not annotate any paralogous relationships. Furthermore, OMA was constructed to identify orthologous pairs; therefore, it is not structured to support evaluations of domain arrangements, also known as domain architectures. At least one other database has been crafted as a multi-domain benchmark. Song et al. manually curated a benchmark of twenty well-studied families in the human and mouse genomes [15]. Drawing on the literature to justify homology, they assembled an initial release that included 1577 sequences from SwissProt, and have since provided an update totaling 1832 sequences. While the Song et al. database is a useful resource for evaluating performance in human and mouse proteins, it also precludes benchmarking the harder challenge of identifying homology among phylogenetically divergent sequences. Finally, Saripella, Sonnhammer, and Forslund constructed three multi-domain databases to evaluate profile-based tools [16]. However, they limited their analysis to strictly non-iterative searching and only used single-domain queries.

Central to assessment of sequence searching tools is the evaluation metric. For the past two decades, the normalized area under a receiver operating characteristic curve (up to *n* false positive records) (ROC _*n*_) [17] has been the primary measure of retrieval of sequence searching tools. To evaluate multiple datasets, some researchers have “pooled” retrievals, sorting all of the records based on their statistical score [9, 16, 18]. This is problematic, in that the records from a single retrieval can dominate the overall area under the curve [19, 20]. We evaluated retrieval with the Threshold Average Precision-*k* (TAP-*k*) metric [20]. In the TAP-*k*, “*k*” imposes a threshold to fix the median number of irrelevant (“false positive”) records per query. This threshold is applied to all the queries. The TAP-*k* is based on the average precision (a standard measure in text retrieval):

1 $$ \frac{1}{T_{q}}\sum\limits_{m=1}^{j\left(E_{0}\right)}p(m)  $$

Here, *T*_*q*_ is the total number of relevant records for a query *q*, *j*(*E*_0_) is the rank of the last relevant record with a statistical score of *E*_0_ or lower and *p*(*x*) is the precision of the record at rank *x*. Notice that there could be irrelevant records with a score lower than *E*_0_ (which reduces the utility of the retrieval) but do not affect in the average precision. The TAP-*k* remedies this situation by penalizing irrelevant records occurring before the threshold *E*_0_ and normalizing to account for the extra precision term:

2 $$ \frac{1}{T_{q} + 1}\left[p\left(E_{0}\right) + \sum\limits_{m=1}^{j\left(E_{0}\right)}p(m)\right]  $$

Due to the normalization, TAP-*k* scores are in the range of 0.0 to 1.0. A TAP-*k* score is 0.0 if no relevant records are retrieved before the cutoff. Conversely, a TAP-*k* score is 1.0 when all the relevant records and no others are retrieved before the cutoff.

In this study, we introduce MultiDomainBenchmark (MDB), the first phylogenetically-broad database retrieval benchmark with multi-domain queries. We anticipate that the primary use of this benchmark will be to evaluate the retrieval performance of searching tools. Namely, the MDB will allow for assessments using multi-domain sequences. Along those lines, and to illustrate the utility of MDB, we benchmarked two sequence searching tools, BLAST/PSI-BLAST [21, 22] and HMMER [23], and list their TAP-*k* and timing performance results here. To determine relevancy, we use a novel approach that accounts for the domain architecture within a protein.

To illustrate the importance of accounting for multiple domains when using a searching tool, we constructed single-domain queries and database from our multi-domain database by creating a new sequence for each domain and its flanking amino acids up to the next domain (or edge of the sequence). While we could use dozens of examples that illustrate the same point, we arbitrarily choose up |Q1L5Y1 |Q1L5Y1_9FILI (GenBank: AAY89355.1, 836 AA) as the query and up |Q76IJ5 |Q76IJ5_9FUNG (GenBank: BAD02841.1, 352 AA) as the target. The query has three domains: PF00623, PF04983 and PF04998. The target has four domains: PF00623, PF04983, PF05000 and PF04998. Using each of the three (single-domain) sequences from the original multi-domain query, we searched using PSI-BLAST against the 337,199 single-domain sequences. Each of the searches listed a hit for the correct single-domain sequence from up |Q76IJ5 |Q76IJ5_FUNG, however, each of the e-values were above the default cut-off of 0.001 (i.e., 0.33, 0.003 and 10, respectively). Conversely, we when search with PSI-BLAST, using the original multiple-domain sequence as a query, it lists the match to up |Q76IJ5 |Q76IJ5_9FUNG with an e-value of 2*e*−18.

## Benchmark construction and content

**Fig. 1:**
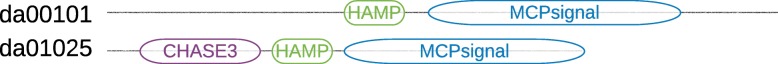
Domain Architecture (DA) examples. Both DAs have protein domains HAMP and MCPsignal, whereas only da01025 has CHASE3. When a sequence from da00101 is used as the query and retrieves a sequence from da01025, we classify the match as relevant (a “true positive”). Conversely, if a sequence from da01025 is the query and retrieves a sequence from da00101, the match does not fully recover the domain structures and therefore we classify it as irrelevant (a “false positive”)

In MultiDomainBenchmark, each multi-domain sequence is cataloged by its domain architecture (DA). We define a DA as an ordered set of domains (i.e., as a vector whose coordinates are domain names, possibly with repetition). Furthermore, we use DAs to perform classification. As a theoretical example, let sequenceA have DA (d_1_, d_2_, d_3_) and sequenceB have DA (d_1_, d_3_, d_2_). Here, although the sequences contain the same domains, the domains appear in a different order. Consequently, each sequence has a different DA and therefore, we classify the match of sequenceA and sequenceB in a retrieval list as irrelevant (a “false positive”). As another example, pfam21 |Q3GCI4 |Q3GCI4_9FIRM (RefSeq WP_011640391) contains the HAMP domain and the MCPsignal domain. These domains, in this order, constitute da00101 (i.e., domain architecture 101) (see Fig. 1). Additionally, up |Q4KE98 |Q4KE98_PSEF5 (RefSeq WP_011060626.1) contains these same two domains (in the same order) and starts with the CHASE3 domain. These three domains, in this order, constitute domain architecture da01025 (see Fig. 1). We define relevancy as follows: if the search query is a sequence with da00101 (e.g., Q3GCI4_9FIRM) and it matches a sequence with da01025 (e.g., Q4KE98_PSEF5), then the searching tool captured the domain architecture, so we classify the match as relevant (a “true positive”). Conversely, if the query has da01025 and the searching tool returns a match that has da00101, then the searching tool has not fully captured the domain architecture and the match therefore is classified as irrelevant. Our definition of relevancy accords with definitions elsewhere, such as in Apic, Gough and Teichmann [24], who note the conservation of the N- to C-terminal ordering of two domains (see also [16, 25]). Other researchers also exploit the concept of ordered set of domains to categorize and analyze protein sequences. Kummerfeld and Teichmann [26] studied the order of domains using directed graphs and found several statistically significant features across many genomes. Additionally, some similarity searching algorithms perform alignments using the ordered sets of domains (“domain arrangements”) to significantly reduce the number of comparisons [14].

**Fig. 2:**
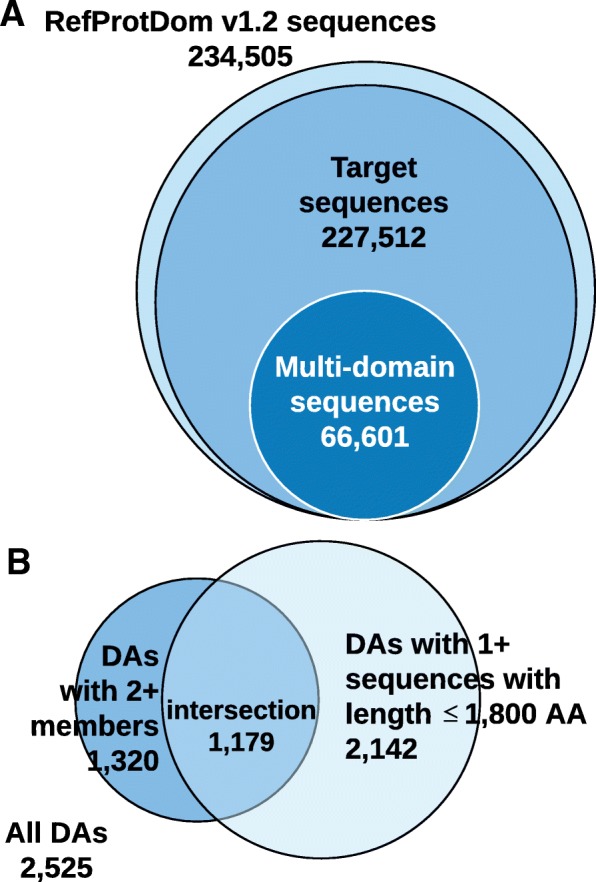
Filtering steps applied to achieve MultiDomainBenchmark. **a** We started with RefProtDom v1.2, then filtered out sequences that had overlapping domain locations. Additionally, we partitioned out the multi-domain sequences. **b** Filtering steps applied to the Domain Architectures (DAs). We started with 2525 DAs, but only considered DAs that had at least one sequence with length ≤1800 amino acids (shown in light blue) and at least two protein sequences (shown in dark blue). The result was 1179 DAs (the intersection)

We created MDB to evaluate genetic database retrieval under realistic conditions, namely, ones using multi-domain queries. Stemming from our familiarity with the curation of the RefProtDom benchmark, we applied several additional filtering steps to RefProtDom and some novel classification concepts to produce MDB. As a starting point, RefProtDom v1.2 has 234,505 sequences. First, we ignored each sequence that had one or more amino acids with multiple domain annotations. Current evaluation measures assume that each amino acid belongs to at most one protein domain. We removed the 6993 sequences with overlapping domains to simplify analyses. We formed the target (or subject) database from the resulting 227,512 (single- and multi-domain) sequences (see Fig. 2a). Next, we excluded the 160,911 sequences that only have one domain, leaving 66,601 multi-domain sequences. For each of the multi-domain sequences, we identified which DA it has (based on its ordered set of domains). Due to variance in the number of repeated domains, we “collapsed” multiple adjacent labels of the same domain into a single instance in the DA [3, 16, 27, 28]. For example, a protein with domains d_1_, d_2_, d_2_, d_2_, d_3_, d_2_ would have a DA of d_1_, d_2_, d_3_, d_2_. We sorted the sequences based on the number of domains (counting collapsed domains as a single domain). We assigned a new (ascending) number to the first occurrence of each DA. In all, there are 2525 unique DAs among the multi-domain sequences (with 32.0% having collapsed domains).

We applied additional filters to the set of DAs before selecting query sequences. First, because we were developing a benchmark, we only considered DAs that had more than one protein sequence with that DA (a DA member) (again simplifying retrieval analyses). Second, we filtered out DAs that did not have at least one sequence shorter or equal to 1800 amino acids (to reduce execution time). This resulted in 1179 DAs (see Fig. 2b). Furthermore, to provide a phylogenetically-broad benchmark, we only considered DAs with sequences in more than one kingdom of life (i.e., Eukarya, Bacteria, Archaea). From each of the remaining 412 DAs, we randomly chose a representative query sequence with length ≤1800 amino acids. We then ordered the queries (by their DA index) and designated the 206 odd ranked queries for the Training set and the 206 even ranked queries for the Test set.

**Fig. 3:**
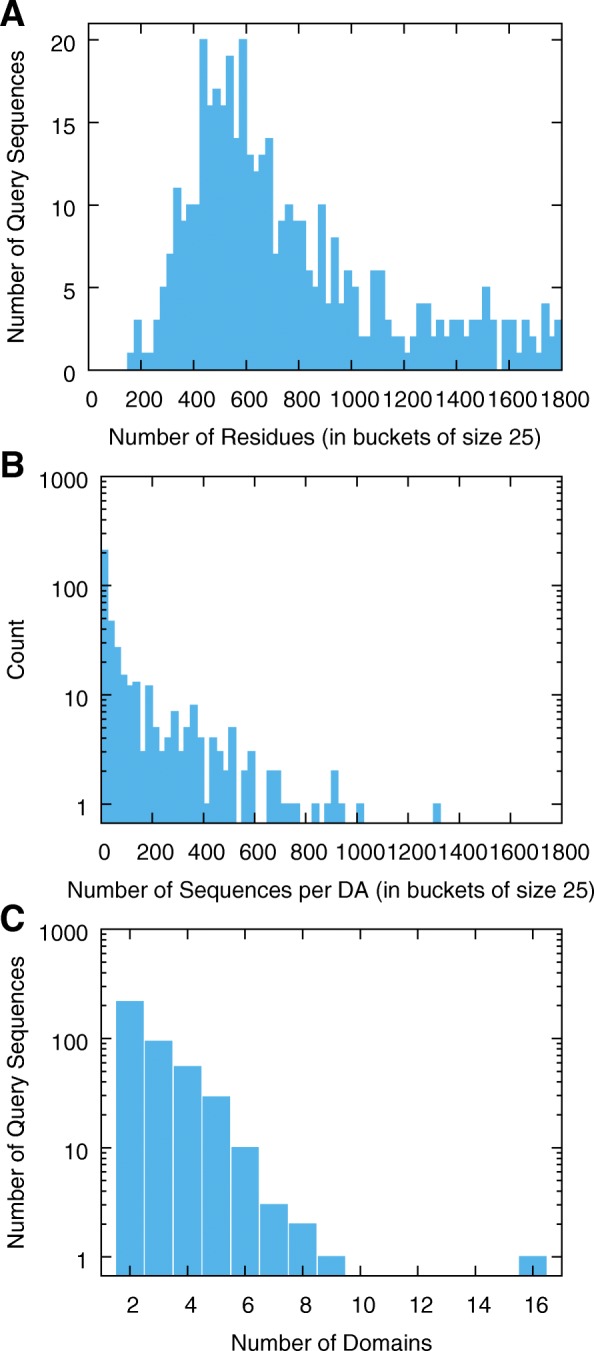
**a** Histogram of the length of all query sequences. For example, there are 20 query sequences that have between 425 and 449 amino acids. **b** Histogram of the number of sequences with the same Domain Architecture (DA). For example, there are three domain architectures that have between 575 and 599 sequences. Note, the y-axis is logarithmic. **c** Distribution of the number of protein domains in the query sequences (after collapsing repeated domain labels). Note, the y-axis is logarithmic

The sequences and DAs in the MDB can be characterized by 1) length of each query sequence, 2) number of sequences in each DA and 3) number of domains per sequences. First, the query sequences range from 170 to 1800 residues long (with an average of 759.7 residues). Figure 3a aggregates all query sequence lengths in a histogram. Second, by requirement of our filtering pipeline, each DA must have at least two sequences. While the largest DA has 1315 sequences, the average number of sequences (per DA) is 111.0 and the median is 23.5. Figure 3b is a histogram indicating the distribution of the number of sequences per DA. Third, while one of the queries has sixteen domains, most queries have two domains (the minimum number) (for an average of 2.9 domains per query sequence). Figure 3c summarizes the number of domains for each of the queries.

As is common with sequence searching benchmarks, the data are contained in flat-text files (readable by any text editor). The target sequences (which include the query sequences) are in a FASTA formatted file. Domain locations and relevancy information are contained in tab-delimited files.

## Utility and discussion

With the explosion of sequence data and more sophisticated tools than ever before, we now have more annotated sequences and genomes available. Multiple databases now include domain annotations (e.g., SCOP, Pfam, CDD [29]). For example, of the sequences with annotated domains in the UniProt-SwissProt database [30], 45.1% have multiple domains with the average number of domains of 4.2 per entry (see Additional file 1 for more details). Although this has led to more discoveries about and emphasis on domains and their role in structure, function and evolution [31], evaluation of searching tools has focused on single domains.

Several derivative works of the Pfam database exist, with RefProtDom being of special interest. RefProtDom applies several additional filters to the Pfam database to create a homology evaluation benchmark. Although RefProtDom version 2 has been released [32], it did not include domain location information, forcing us to use version 1.2.

Relevancy is more clearly defined for single domain matches. Consequently, if a researcher is primarily concerned with just a single domain, then the results of the evaluation of searching tools using existing single-domain benchmarks are probably adequate for that use case. If however, the protein(s) of interest have multiple domains or are being compared against multi-domain proteins, then the evaluation results from a multi-domain benchmark may prove more valuable. Furthermore, although many protocols for manipulating domain architectures collapse adjacent repeated domains into one, the consequences of the collapse are not fully understood. Researchers exploring the relevancy of retrieved proteins with repeated domains should therefore inspect the corresponding results carefully. Finally, most search tools do not try to detect domain rearrangements. Accordingly, we do not try to capture domain rearrangements with this benchmark.

Although other multi-domain databases and benchmarks do exist, they are not structured as general-purpose benchmarks. For example, the gold-standard benchmark introduced by Song et al. is noticeably different from MultiDomainBenchmark. First, it only comprises human and mouse sequences. Second, it is much smaller with only 0.8% of the number of sequences in MDB (and therefore fewer relationships defined).

On one hand, MultiDomainBenchmark places heavy restrictions on domain architecture, namely, it insists that retrieved proteins should match all query domains, matching the query order though not the multiplicity (because it collapses multiple domains into one). On the other hand, many domain benchmarks count a single domain match as correct, while yet others could count multiple domain matches with omissions as correct. The difference reflects the intent of MultiDomainBenchmark: to evaluate tools for retrieving proteins whose functions overlap very tightly with the query protein.

Consider for example, the inhibitor of apoptosis (IAP) family, whose members c-IAP1 and c-IAP2 contain the domain architectures BIR-BIR-BIR-UBA-CARD-RING, and whose member XIAP contains the slightly different architecture BIR-BIR-BIR-UBA-RING, omitting the CARD domain. For the query c-IAP2, most domain benchmarks would count both c-IAP1 and XIAP as correct hits, whereas MultiDomainBenchmark insists on a more precise structural overlap, so with query c-IAP2 it would count c-IAP1 as a correct hit, but not XIAP.

### Case study: sequence searching tool evaluations

**Fig. 4:**
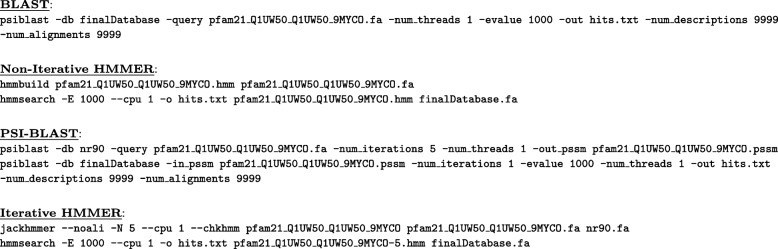
Abbreviated command-line examples for non-iterative searches. For BLAST, we searched with PSI-BLAST set to a single iteration on the MultiDomainBenchmark target database. For non-iterative HMMER, we first produced a hidden Markov model (HMM) with hmmbuild, then searched the MDB target database using that HMM with hmmsearch. For PSI-BLAST, first, we search for up to five iterations on a clustered version of the NR database (see main text for details), saving the resulting position-specific scoring matrix (PSSM). Then, using the resulting PSSM, we searched the MDB target database. For iterative HMMER, we saved the resulting HMM produced by searching up to five iterations with jackhmmer. Then, we performed a final search on the MDB target database with hmmsearch using the resulting HMM. The e-value threshold (and -num_descriptions and -num_alignments) were set artificially high for performance analysis reasons. For complete command-line usage, see the MDB website

Because of their widespread use, we chose two sequence searching tools to illustrate the usefulness of MultiDomainBenchmark: BLAST/PSI-BLAST and HMMER. We evaluated each tool with both non-iterative and iterative protocols. For non-iterative evaluations, we searched against the collection of 227,512 sequences (with non-overlapping domains) in the MDB target database using each of the 206 MDB Test queries. Figure 4 provides command-line examples for one of the queries for both BLAST and non-iterative HMMER. For iterative evaluations, we first performed up to five rounds of searching on a clustered version of NCBI’s NR database [33]. We clustered the NR database at 90% redundancy using nrdb90.pl [34] to reduce its size for execution time considerations per industry standard [10, 35]. A final search was performed on the MDB target database, with the profile built from the iterative rounds. We executed each of the sequence searching tools with most of the default arguments, except to specify the query, database and number of iterations and output files. Figure 4 provides command-line examples for one of the Test queries for both PSI-BLAST and iterative HMMER.

Due to ambiguities inherent with classifying the homology of multi-domain searches, we focused instead on capturing domain architectures. In addition to the criterion for a match to be classified as relevant (a “true positive”) described in the “Benchmark construction and content” section (i.e., query and target sequences having the same domains in the same order), we added an additional constraint. The relevancy scoring also required that at least 50% coverage [36] (i.e., the alignment identified by the tool must correspond to 50% or more of the amino acids within the annotated boundaries of the domains). All other matches were classified as irrelevant (“false positives”). This additional constraint ensures that the tool has guided the researcher to the correct portion of the protein to identify the domain architecture. If a tool does not accurately identify the correct alignment, then it has merely made a lucky guess. We evaluated retrieval with the Threshold Average Precision-*k* (TAP-*k*) metric [20].

**Fig. 5:**
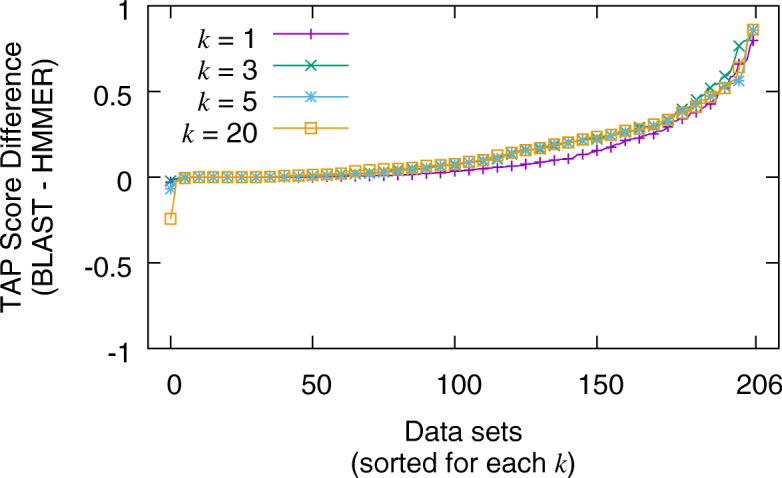
Distribution of differences in non-iterative TAP-*k* scores (for *k*={1,3,5,20}) between BLAST and HMMER for the MultiDomainBenchmark Test queries. The average differences (and standard deviations) are 0.12 ±0.18, 0.16 ±0.20, 0.15 ±0.18 and 0.16 ±0.18 for *k*={1,3,5,20} respectively. A larger area under the curve indicates that BLAST had more datasets that performed better. Note, the x-axis is sorted independently for each *k*

**Fig. 6:**
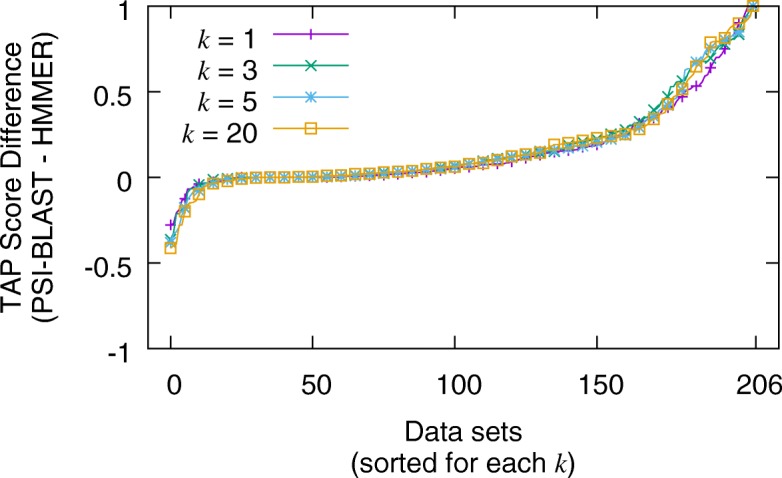
Distribution of differences in iterative TAP-*k* scores (for *k*={1,3,5,20}) between PSI-BLAST and HMMER for the MultiDomainBenchmark Test queries (using the profile generated from searching up to five iterations on a clustered version of the NR database). The average differences (and standard deviations) are 0.16 ±0.25, 0.18 ±0.27, 0.17 ±0.27 and 0.17 ±0.27 for *k*={1,3,5,20} respectively. A larger area under the curve indicates that PSI-BLAST had more datasets that performed better. Note, the x-axis is sorted independently for each *k*

Given the phylogenetically diverse set of queries in the MDB Test subset, the TAP-*k* scores for both searching tools span the full range from 0.0 to 1.0. Figure 5 summarizes the results for the non-iterative search executions by plotting the difference of subtracting HMMER’s TAP-*k* scores from BLAST’s for each data set (larger values indicate BLAST performed better than HMMER). Note, for each value of *k*={1,3,5,20}, the x-axis is sorted independently to provide a visually discernible graph. The most common difference is exactly 0.0, as one might expect. For *k*=20, 7.2% of the TAP scores were the same. This percentage increases as *k* decreases with *k*=1 having 19.4% of its scores being the same. The average difference varies from 0.12 (for *k*=1), to 0.16 (for *k*=3) (larger averages indicate BLAST performed better than HMMER). Figure 6 summarizes the results for the iterative search executions by plotting the differences for subtracting HMMER from PSI-BLAST (larger values indicate PSI-BLAST performed better than HMMER). Here, TAP-*k* scores for iterative searches show much more discord than for the non-iterative ones. For example, the percentage of searches that have the same TAP-*k* score varied from 3.8% (*k*=20) to 15.0% (*k*=1). Additionally, the averages ranged from 0.16 (*k*=1) to 0.18 (*k*=3). The distribution of TAP-*k* scores is illustrated in the Additional file 1.

**Fig. 7:**
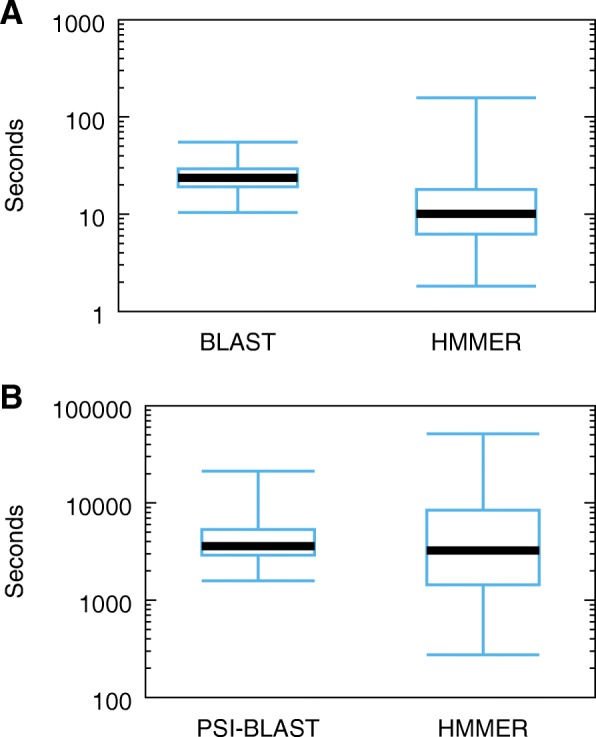
Box-and-whisker plot of the non-iterative (**a**) and iterative (**b**) execution times for BLAST/PSI-BLAST and HMMER (non-iterative: hmmsearch; iterative: jackhmmer + hmmsearch) for the MultiDomainBenchmark Test queries. Whiskers represent the shortest and longest execution times. The blue box indicates the first and third quartiles and the thick black line the second quartile (or median)

Additionally, we gathered timing results. We executed the programs on a shared environment system and therefore the timing results are just first approximations to the actual execution times. Figure 7 summarizes the timing results for BLAST/PSI-BLAST and HMMER using box-and-whisker plots. The whiskers represent the minimum and maximum execution times. The bottom and the top of the (blue) box in each plot indicate the first and third quartiles. The thick black horizontal line represents the second quartile (or median) value. Note, the y-axis is logarithmic. For the non-iterative runs, HMMER generally has faster execution times than BLAST with a median of 10 s compared to BLAST’s 24 s. For the iterative runs, PSI-BLAST’s median is one hour and 0 min compared to HMMER’s median execution time of 54 min (however, PSI-BLAST’s average is one hour and 19 min compared to HMMER’s average execution time of one hour and 37 min).

**Fig. 8:**
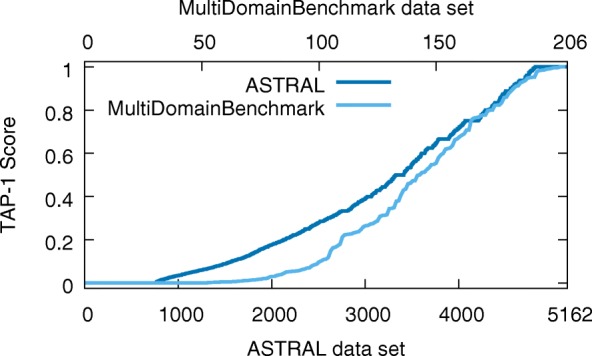
PSI-BLAST TAP-1 scores for both the (single-domain) ASTRAL database (bottom x-axis) and MultiDomainBenchmark (top x-axis). These two distributions have a *p*-value of 0.0114 of being from the same population (see the main text for details)

Researchers have been benchmarking sequence searching tools for decades. With just the exceptions mentioned previously, these benchmarks have only had single-domain sequences. As one would expect, sequence searching tools perform differently on single- and multi-domain benchmarks. To quantify this, we divided the ASTRAL database into two halves, each with 5162 sequences (as has been done elsewhere [11]). We compared the distribution of TAP-1 scores for PSI-BLAST on ASTRAL and MDB (see Fig. 8). The average PSI-BLAST TAP-1 score on the ASTRAL database is 0.38 whereas the average on the MDB is 0.33. Using a one-sided Wilcoxon-Mann-Whitney test [37], the probability that the two distributions of scores coming from the same population is *p*=0.0114.

## Conclusion

In this study, we presented MultiDomainBenchmark, the first phylogenetically diverse benchmark with multi-domain queries. MDB has a target database with 227,512 single- and multi-domain sequences. The 66,602 multi-domain sequences have 2525 unique DAs. We applied additional filters yielding 412 phylogenetically diverse DAs and from each one we randomly selected a query sequence. We designed this benchmark on the one hand, to bring attention to the issue of evaluation of searches with multiple domains, and on the other, to perform such analyses. Here, we also provided the initial use of MDB by assessing BLAST/PSI-BLAST’s and HMMER’s ability to capture domain architectures and their execution times. While many other sequence searching tool exist, our case study here simply demonstrates the use of MDB.

We invite other developers and researchers to also use MDB. To this end (and for reproducibility), we provide the scripts on our website that we used to perform the case study.

## Additional file

Additional file 1 Supplementary material. Supplementary material detailing multi-domain proteins in UniProt-SwissProt and the distribution of TAP-*k* scores from the case study. (PDF 259 kb)

## Abbreviations

DA: Domain architecture
HMM: Hidden Markov model
OMA: Orthologous MAtrix
PSSM: position-specific scoring matrix
ROC: receiver operating characteristic
TAP: Threshold average precision
