# Supplementary material for: MultiDomainBenchmark: a multi-domain query and subject database suite
Source: BMC Bioinformatics. 2019 Feb 14;20:77. doi: 10.1186/s12859-019-2660-5 (PMC6376684; doi:10.1186/s12859-019-2660-5)
Supplement: Supplementary file 1 — Supplementary material. Supplementary material detailing multi-domain proteins in UniProt-SwissProt and the distribution of TAP-k scores from the case study. (PDF 259 kb) [file 12859_2019_2660_MOESM1_ESM.pdf]

MultiDomainBenchmark: A Multi-domain Query and Subject Database Suite: Supplementary Material

Hyrum D. Carroll [1], John L. Spouge [2] and Mileidy Gonzalez [2]

[1]TSYS School of Computer Science, Columbus State University, Columbus, GA, 31907, USA,
[2]National Center for Biotechnology Information, Bethesda, MD 20894, USA

1 MULTI-DOMAINS IN UNIPROT-SWISSPROT

The UniProt-SwissProt database (downloaded February 27, 2018) has 556,567 sequences. 137,997 of those sequences have a DOMAIN and/or TOPO_DOM Feature Table annotation. 60,311 (or 43.7%) of those sequences have more than one DOMAIN and/or TOPO_DOM annotation, illustrating the prevalence of multi-domain sequences. Of the sequences with DOMAIN and/or TOPO_DOM annotations, the average number of domains is 2.4. For the multi-domain sequences, the average is 4.2. Figure 1 details the distribution of the number of domains per entry.

2 DISTRIBUTION OF TAP-K SCORES

Figure 2 illustrates the the distribution of TAP-k scores for the non-iterative results and Figure 3 iterative results from the Case Study (see the main paper for background details). In each box-and-whisker plot, the whiskers represent the best and worst TAP-k score. Given the broad range of queries, each plot has scores of 0.0. In fact, the lowest number of 0.0 scores is for $k = 1$ for non-iterative HMMER (with 32 scores of 0.0). The most number of 0.0 scores is for $k = 1$ for iterative HMMER (with 92 scores of 0.0). All of the plots include at least one score of 1.0, with the exception of $k = 20$ for iterative HMMER (which has a highest score of 0.99). In each plot, the blue box indicates the first and third quartiles and the thick black line the second quartile (or median).

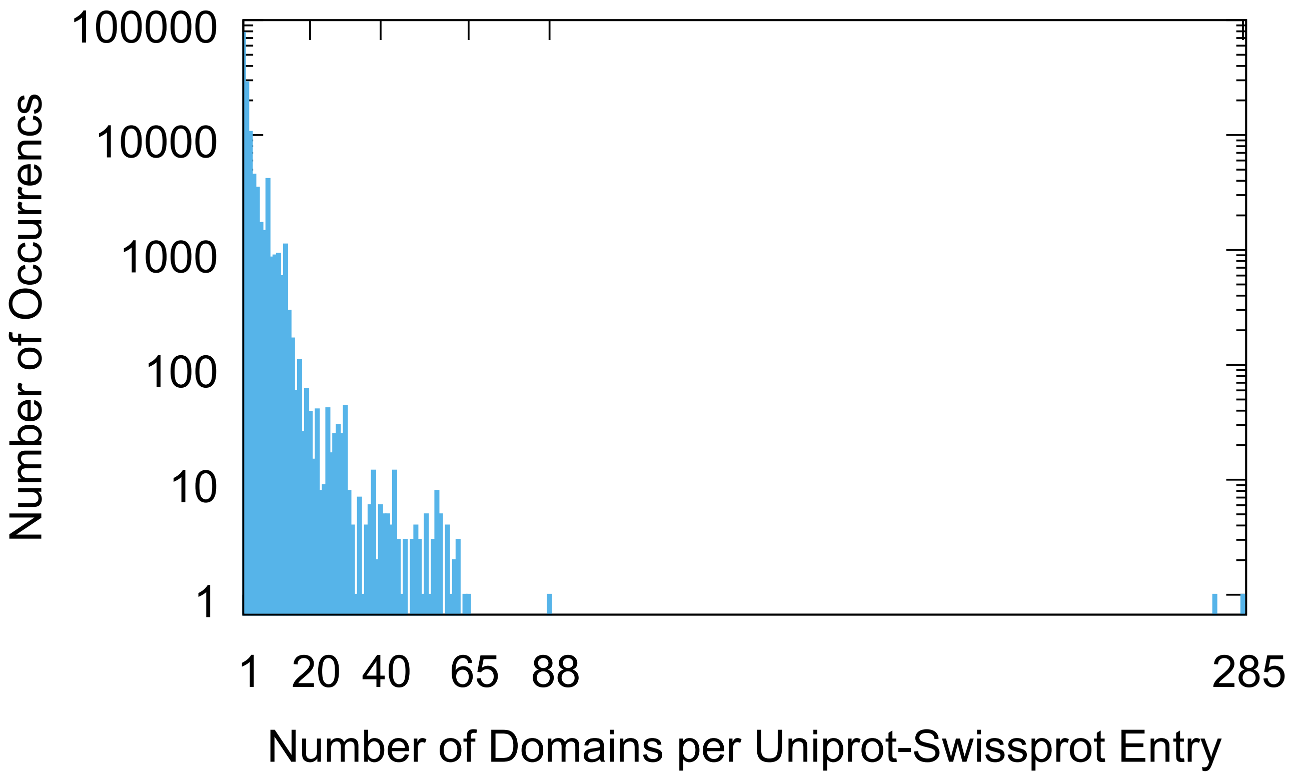

Fig. 1. Histogram of the number of domains per Uniprot-Swissprot entry. Note, the y-axis is logarithmic.

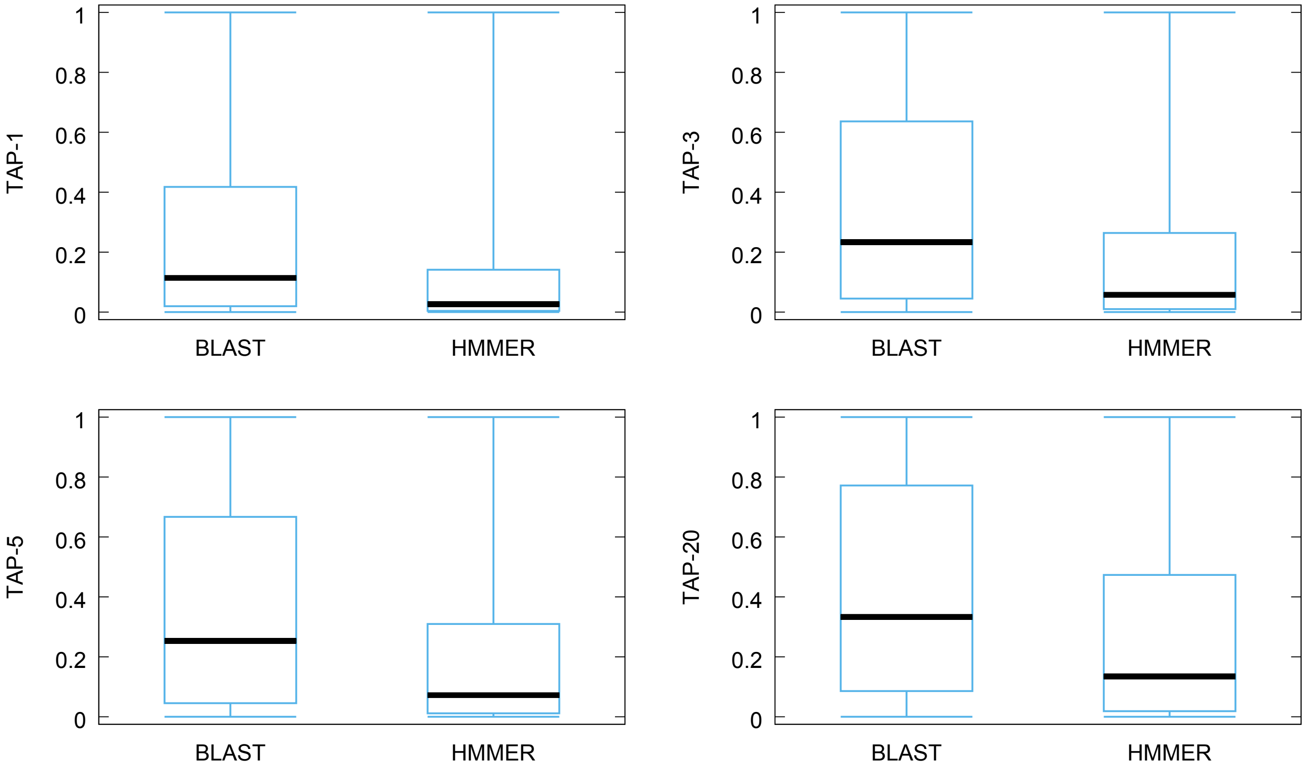

Fig. 2. Box-and-whisker plots of the non-iterative TAP-{1,3,5,20} results of BLAST and HMMER for the MultiDomainBenchmark Test queries, searching against the MultiDomainBenchmark target database. The whiskers represent the maximum and minimum TAP scores. The blue box indicates the first and third quartiles and the thick black line the second quartile (or median).

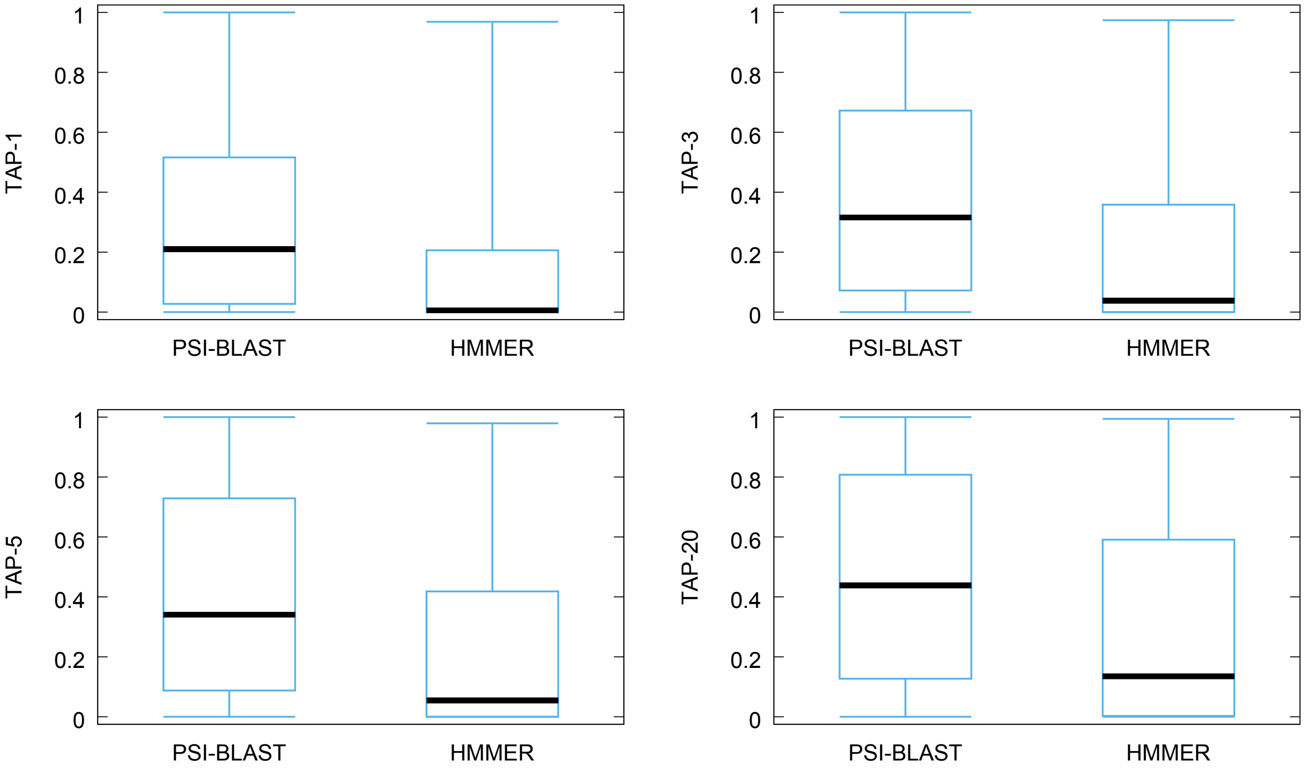

Fig. 3. Box-and-whisker plots of the iterative TAP-{1,3,5,20} results of PSI-BLAST and HMMER for the MultiDomainBenchmark Test queries (using the profile generated from searching up to five iterations on a clustered version of the NR database). The whiskers represent the maximum and minimum TAP scores. The blue box indicates the first and third quartiles and the thick black line the second quartile (or median).
